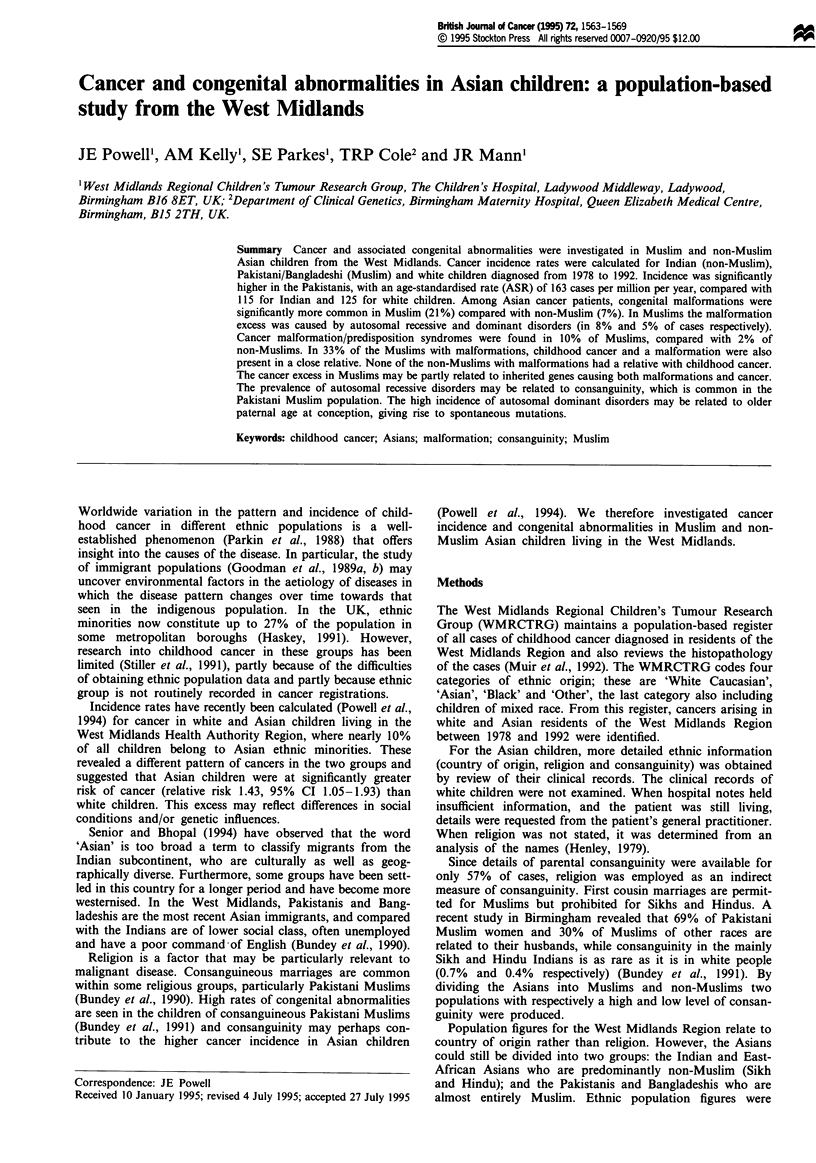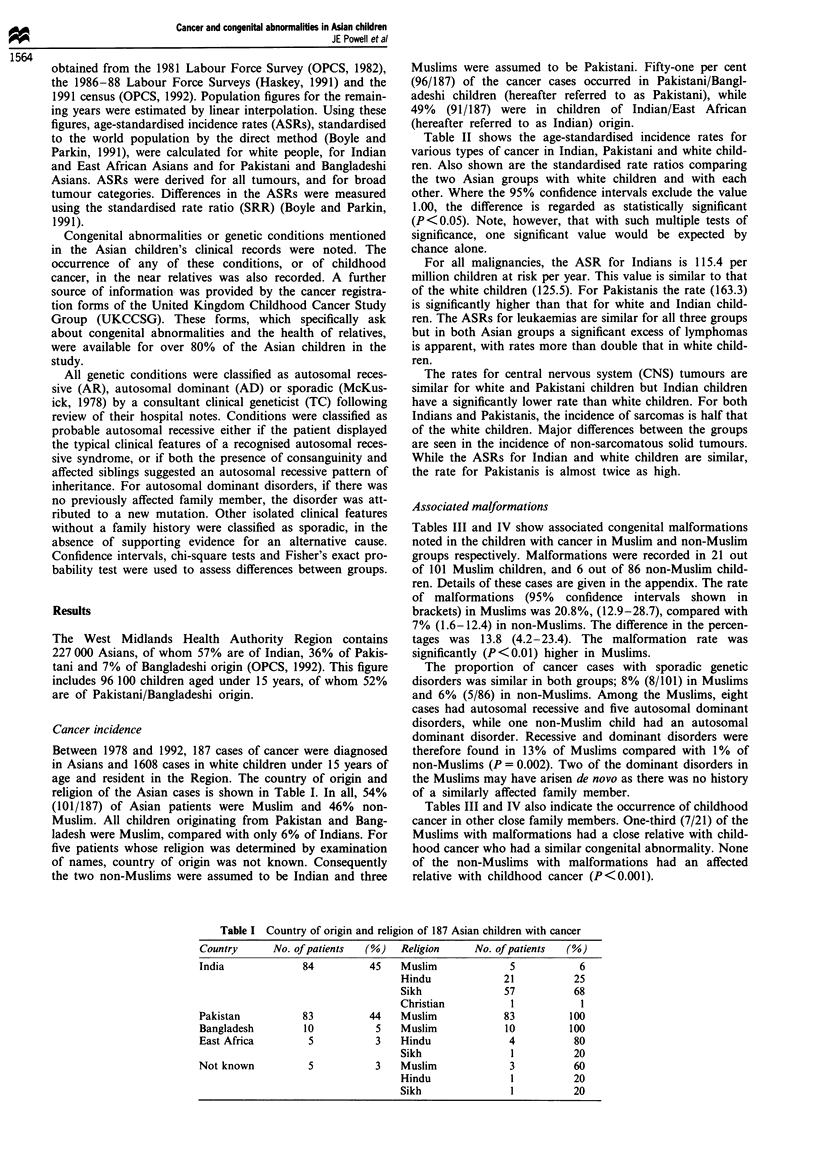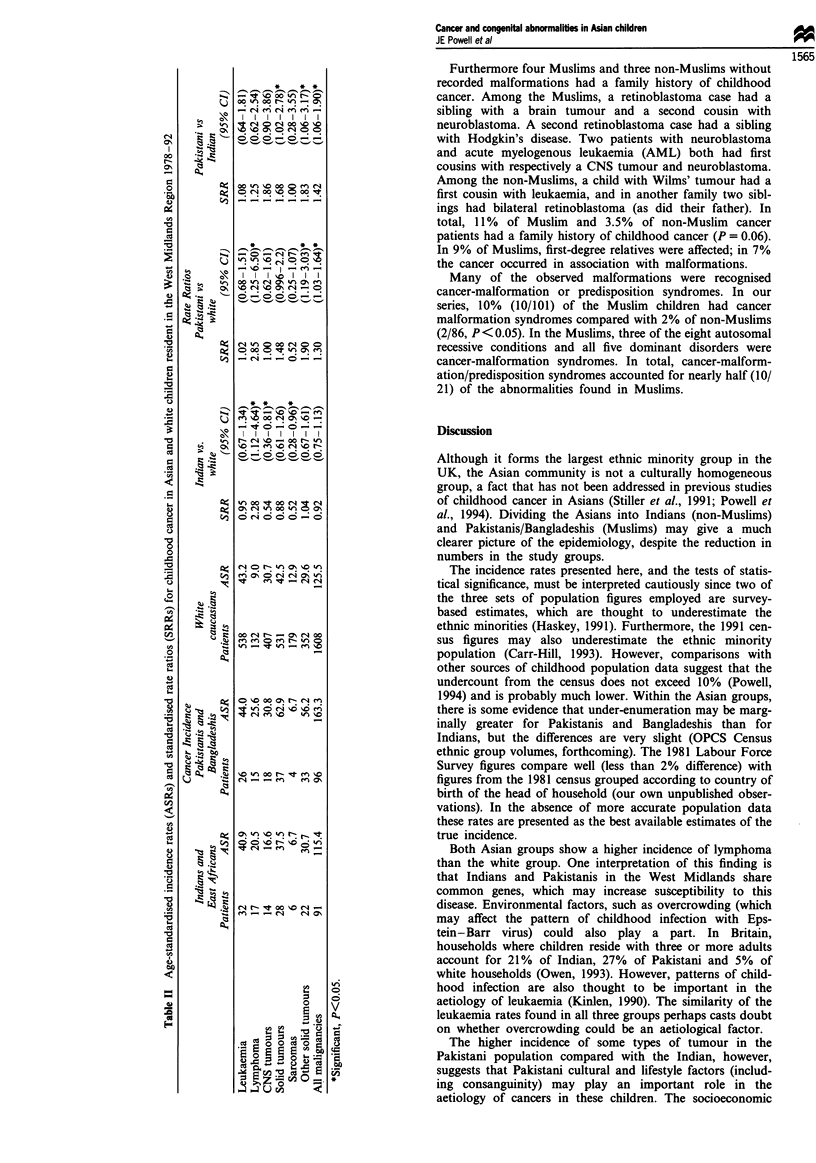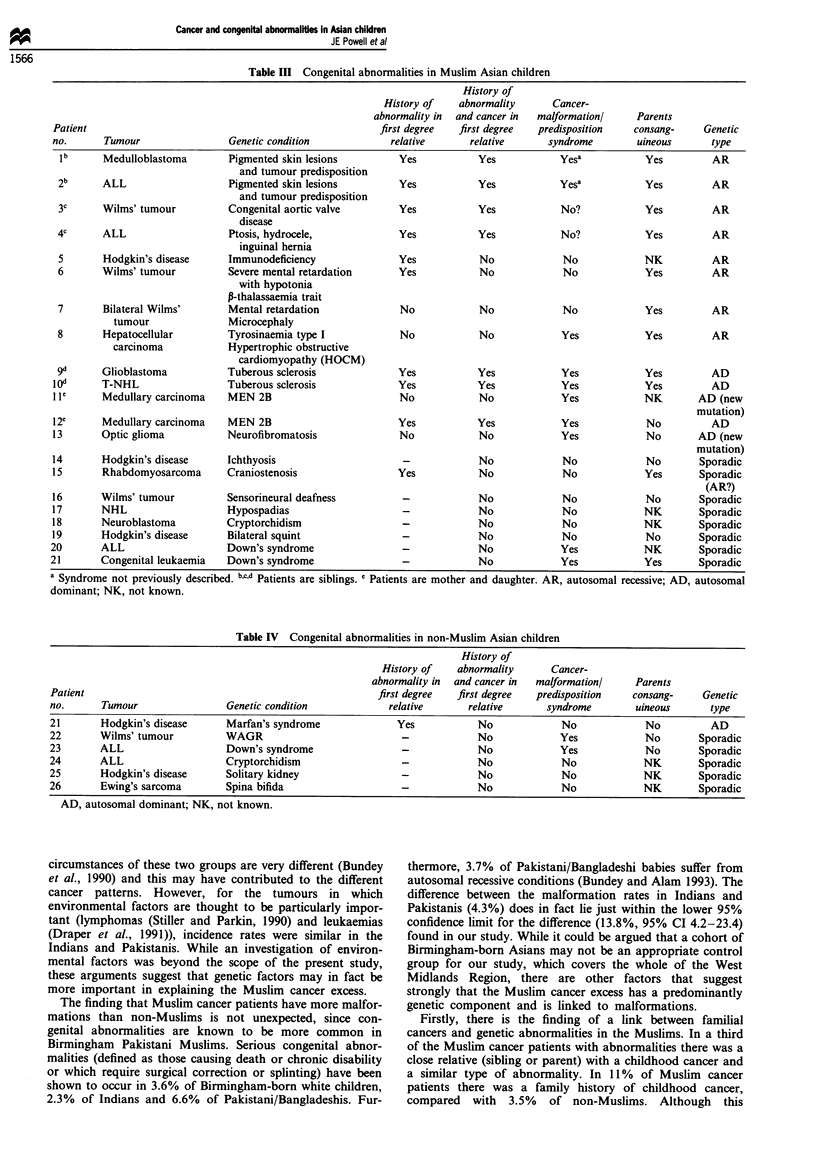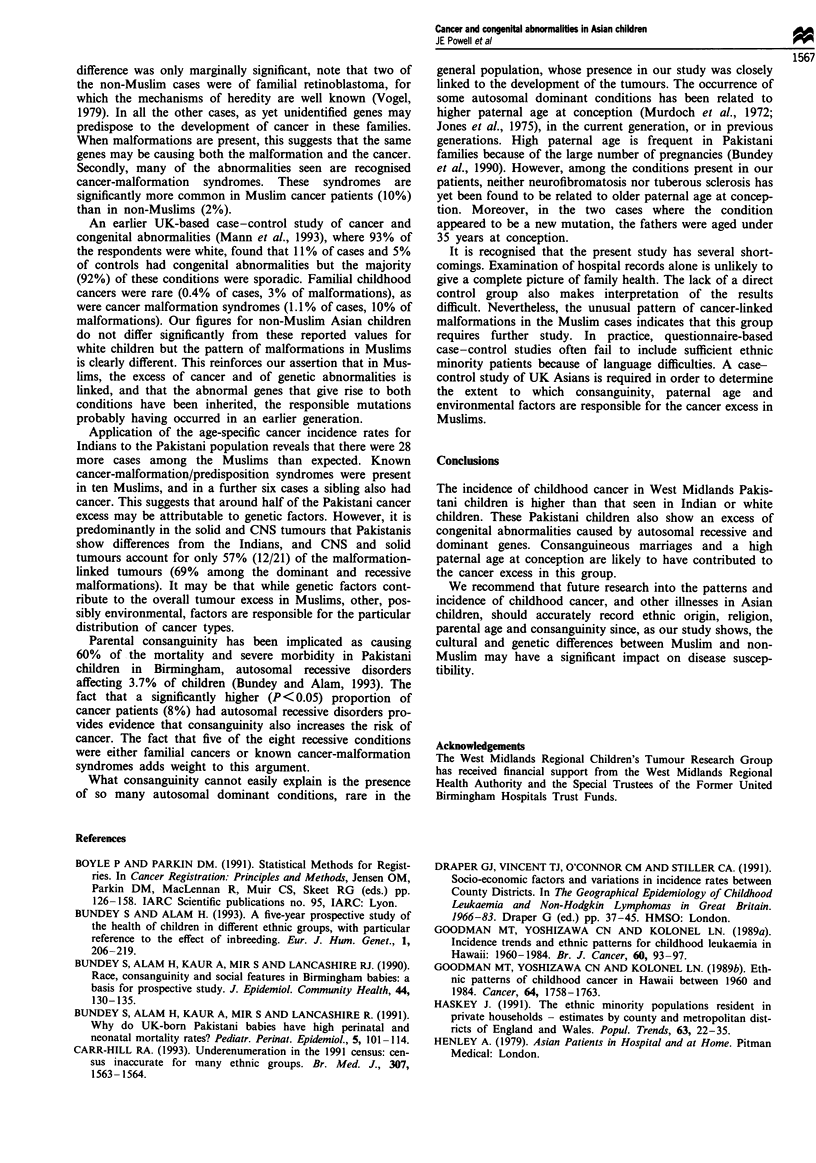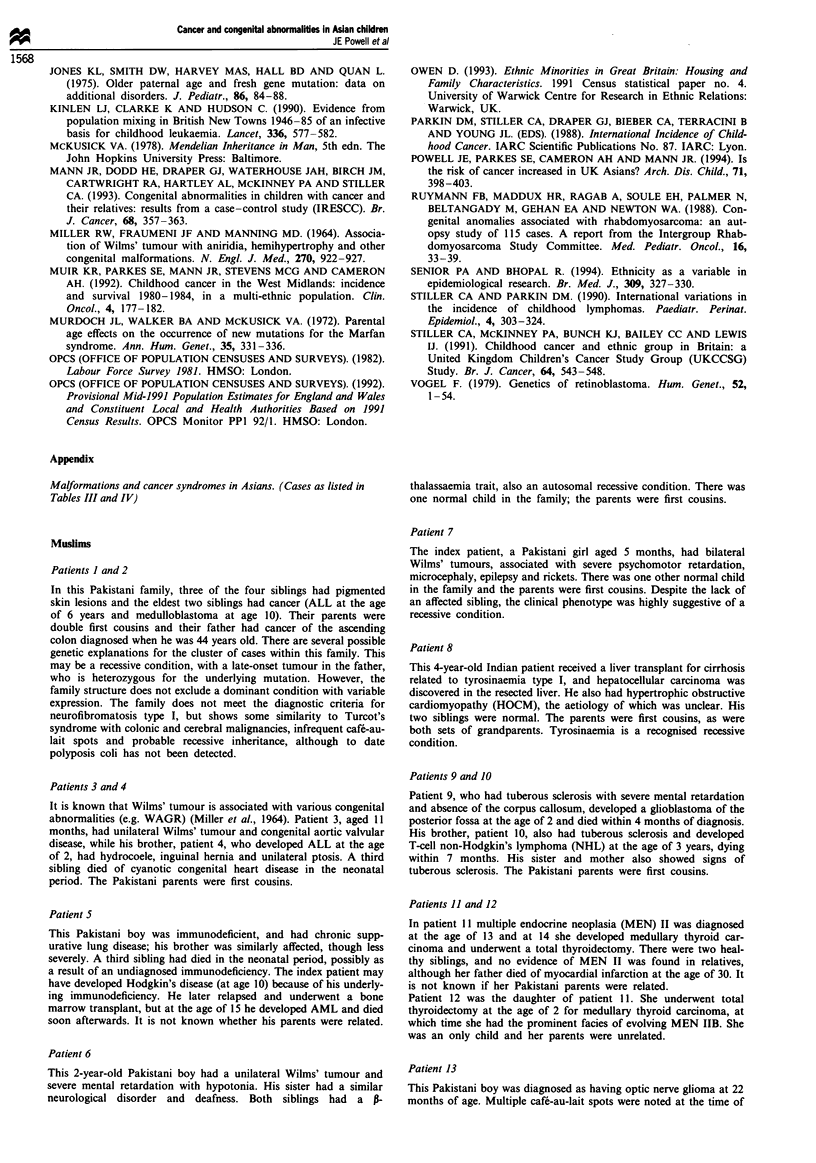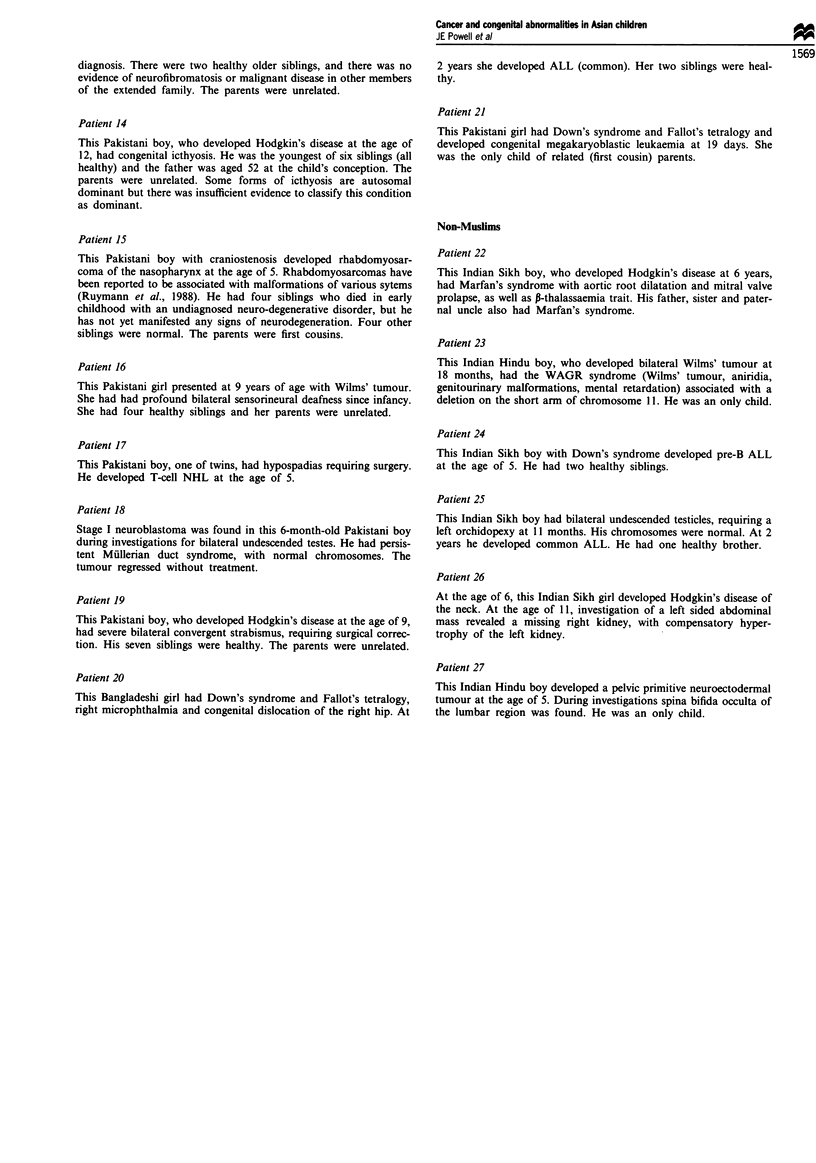# Cancer and congenital abnormalities in Asian children: a population-based study from the West Midlands.

**DOI:** 10.1038/bjc.1995.549

**Published:** 1995-12

**Authors:** J. E. Powell, A. M. Kelly, S. E. Parkes, T. R. Cole, J. R. Mann

**Affiliations:** West Midlands Regional Children's Tumour Research Group, Children's Hospital, Ladywood, Birmingham, UK.

## Abstract

Cancer and associated congenital abnormalities were investigated in Muslim and non-Muslim Asian children from the West Midlands. Cancer incidence rates were calculated for Indian (non-Muslim), Pakistani/Bangladeshi (Muslim) and white children diagnosed from 1978 to 1992. Incidence was significantly higher in the Pakistanis, with an age-standardised rate (ASR) of 163 cases per million per year, compared with 115 for Indian and 125 for white children. Among Asian cancer patients, congenital malformations were significantly more common in Muslim (21%) compared with non-Muslim (7%). In Muslims the malformation excess was caused by autosomal recessive and dominant disorders (in 8% and 5% of cases respectively). Cancer malformation/predisposition syndromes were found in 10% of Muslims, compared with 2% of non-Muslims. In 33% of the Muslims with malformations, childhood cancer and a malformation were also present in a close relative. None of the non-Muslims with malformations had a relative with childhood cancer. The cancer excess in Muslims may be partly related to inherited genes causing both malformations and cancer. The prevalence of autosomal recessive disorders may be related to consanguinity, which is common in the Pakistani Muslim population. The high incidence of autosomal dominant disorders may be related to older paternal age at conception, giving rise to spontaneous mutations.


British Journal of Cancer (1995) 72, 1563-1569
© 1995 Stockton Press All rights reserved 0007-0920/95 $12.00

### Cancer and congenital abnormalities in Asian children: a population-based study from the West Midlands

JE Powell[1], AM Kelly[1], SE Parkes[1], TRP Cole[2] and JR Mann[1]

[1]*West Midlands Regional Children's Tumour Research Group, The Children's Hospital, Ladywood Middleway, Ladywood, Birmingham B16 8ET, UK;* [2]*Department of Clinical Genetics, Birmingham Maternity Hospital, Queen Elizabeth Medical Centre, Birmingham, B15 2TH, UK.*

**Summary** Cancer and associated congenital abnormalities were investigated in Muslim and non-Muslim Asian children from the West Midlands. Cancer incidence rates were calculated for Indian (non-Muslim), Pakistani/Bangladeshi (Muslim) and white children diagnosed from 1978 to 1992. Incidence was significantly higher in the Pakistanis, with an age-standardised rate (ASR) of 163 cases per million per year, compared with 115 for Indian and 125 for white children. Among Asian cancer patients, congenital malformations were significantly more common in Muslim (21%) compared with non-Muslim (7%). In Muslims the malformation excess was caused by autosomal recessive and dominant disorders (in 8% and 5% of cases respectively). Cancer malformation/predisposition syndromes were found in 10% of Muslims, compared with 2% of non-Muslims. In 33% of the Muslims with malformations, childhood cancer and a malformation were also present in a close relative. None of the non-Muslims with malformations had a relative with childhood cancer. The cancer excess in Muslims may be partly related to inherited genes causing both malformations and cancer. The prevalence of autosomal recessive disorders may be related to consanguinity, which is common in the Pakistani Muslim population. The high incidence of autosomal dominant disorders may be related to older paternal age at conception, giving rise to spontaneous mutations.

**Keywords:** childhood cancer; Asians; malformation; consanguinity; Muslim

Worldwide variation in the pattern and incidence of childhood cancer in different ethnic populations is a well-established phenomenon (Parkin *et al.*, 1988) that offers insight into the causes of the disease. In particular, the study of immigrant populations (Goodman *et al.*, 1989*a*, *b*) may uncover environmental factors in the aetiology of diseases in which the disease pattern changes over time towards that seen in the indigenous population. In the UK, ethnic minorities now constitute up to 27% of the population in some metropolitan boroughs (Haskey, 1991). However, research into childhood cancer in these groups has been limited (Stiller *et al.*, 1991), partly because of the difficulties of obtaining ethnic population data and partly because ethnic group is not routinely recorded in cancer registrations.

Incidence rates have recently been calculated (Powell *et al.*, 1994) for cancer in white and Asian children living in the West Midlands Health Authority Region, where nearly 10% of all children belong to Asian ethnic minorities. These revealed a different pattern of cancers in the two groups and suggested that Asian children were at significantly greater risk of cancer (relative risk 1.43, 95% CI 1.05-1.93) than white children. This excess may reflect differences in social conditions and/or genetic influences.

Senior and Bhopal (1994) have observed that the word 'Asian' is too broad a term to classify migrants from the Indian subcontinent, who are culturally as well as geographically diverse. Furthermore, some groups have been settled in this country for a longer period and have become more westernised. In the West Midlands, Pakistanis and Bangladeshis are the most recent Asian immigrants, and compared with the Indians are of lower social class, often unemployed and have a poor command of English (Bundey *et al.*, 1990).

Religion is a factor that may be particularly relevant to malignant disease. Consanguineous marriages are common within some religious groups, particularly Pakistani Muslims (Bundey *et al.*, 1990). High rates of congenital abnormalities are seen in the children of consanguineous Pakistani Muslims (Bundey *et al.*, 1991) and consanguinity may perhaps contribute to the higher cancer incidence in Asian children (Powell *et al.*, 1994). We therefore investigated cancer incidence and congenital abnormalities in Muslim and non-Muslim Asian children living in the West Midlands.

Correspondence: JE Powell
Received 10 January 1995; revised 4 July 1995; accepted 27 July 1995

### Methods

The West Midlands Regional Children's Tumour Research Group (WMRCTRG) maintains a population-based register of all cases of childhood cancer diagnosed in residents of the West Midlands Region and also reviews the histopathology of the cases (Muir *et al.*, 1992). The WMRCTRG codes four categories of ethnic origin; these are 'White Caucasian', 'Asian', 'Black' and 'Other', the last category also including children of mixed race. From this register, cancers arising in white and Asian residents of the West Midlands Region between 1978 and 1992 were identified.

For the Asian children, more detailed ethnic information (country of origin, religion and consanguinity) was obtained by review of their clinical records. The clinical records of white children were not examined. When hospital notes held insufficient information, and the patient was still living, details were requested from the patient's general practitioner. When religion was not stated, it was determined from an analysis of the names (Henley, 1979).

Since details of parental consanguinity were available for only 57% of cases, religion was employed as an indirect measure of consanguinity. First cousin marriages are permitted for Muslims but prohibited for Sikhs and Hindus. A recent study in Birmingham revealed that 69% of Pakistani Muslim women and 30% of Muslims of other races are related to their husbands, while consanguinity in the mainly Sikh and Hindu Indians is as rare as it is in white people (0.7% and 0.4% respectively) (Bundey *et al.*, 1991). By dividing the Asians into Muslims and non-Muslims two populations with respectively a high and low level of consanguinity were produced.

Population figures for the West Midlands Region relate to country of origin rather than religion. However, the Asians could still be divided into two groups: the Indian and East-African Asians who are predominantly non-Muslim (Sikh and Hindu); and the Pakistanis and Bangladeshis who are almost entirely Muslim. Ethnic population figures were obtained from the 1981 Labour Force Survey (OPCS, 1982), the 1986–88 Labour Force Surveys (Haskey, 1991) and the 1991 census (OPCS, 1992). Population figures for the remaining years were estimated by linear interpolation. Using these figures, age-standardised incidence rates (ASRs), standardised to the world population by the direct method (Boyle and Parkin, 1991), were calculated for white people, for Indian and East African Asians and for Pakistani and Bangladeshi Asians. ASRs were derived for all tumours, and for broad tumour categories. Differences in the ASRs were measured using the standardised rate ratio (SRR) (Boyle and Parkin, 1991).

Congenital abnormalities or genetic conditions mentioned in the Asian children's clinical records were noted. The occurrence of any of these conditions, or of childhood cancer, in the near relatives was also recorded. A further source of information was provided by the cancer registration forms of the United Kingdom Childhood Cancer Study Group (UKCCSG). These forms, which specifically ask about congenital abnormalities and the health of relatives, were available for over 80% of the Asian children in the study.

All genetic conditions were classified as autosomal recessive (AR), autosomal dominant (AD) or sporadic (McKusick, 1978) by a consultant clinical geneticist (TC) following review of their hospital notes. Conditions were classified as probable autosomal recessive either if the patient displayed the typical clinical features of a recognised autosomal recessive syndrome, or if both the presence of consanguinity and affected siblings suggested an autosomal recessive pattern of inheritance. For autosomal dominant disorders, if there was no previously affected family member, the disorder was attributed to a new mutation. Other isolated clinical features without a family history were classified as sporadic, in the absence of supporting evidence for an alternative cause. Confidence intervals, chi-square tests and Fisher's exact probability test were used to assess differences between groups.

### Results

The West Midlands Health Authority Region contains 227 000 Asians, of whom 57% are of Indian, 36% of Pakistani and 7% of Bangladeshi origin (OPCS, 1992). This figure includes 96 100 children aged under 15 years, of whom 52% are of Pakistani/Bangladeshi origin.

### *Cancer incidence*

Between 1978 and 1992, 187 cases of cancer were diagnosed in Asians and 1608 cases in white children under 15 years of age and resident in the Region. The country of origin and religion of the Asian cases is shown in Table I. In all, 54% (101/187) of Asian patients were Muslim and 46% non-Muslim. All children originating from Pakistan and Bangladesh were Muslim, compared with only 6% of Indians. For five patients whose religion was determined by examination of names, country of origin was not known. Consequently the two non-Muslims were assumed to be Indian and three Muslims were assumed to be Pakistani. Fifty-one per cent (96/187) of the cancer cases occurred in Pakistani/Bangladeshi children (hereafter referred to as Pakistani), while 49% (91/187) were in children of Indian/East African (hereafter referred to as Indian) origin.

Table II shows the age-standardised incidence rates for various types of cancer in Indian, Pakistani and white children. Also shown are the standardised rate ratios comparing the two Asian groups with white children and with each other. Where the 95% confidence intervals exclude the value 1.00, the difference is regarded as statistically significant ($P<0.05$). Note, however, that with such multiple tests of significance, one significant value would be expected by chance alone.

For all malignancies, the ASR for Indians is 115.4 per million children at risk per year. This value is similar to that of the white children (125.5). For Pakistanis the rate (163.3) is significantly higher than that for white and Indian children. The ASRs for leukaemias are similar for all three groups but in both Asian groups a significant excess of lymphomas is apparent, with rates more than double that in white children.

The rates for central nervous system (CNS) tumours are similar for white and Pakistani children but Indian children have a significantly lower rate than white children. For both Indians and Pakistanis, the incidence of sarcomas is half that of the white children. Major differences between the groups are seen in the incidence of non-sarcomatous solid tumours. While the ASRs for Indian and white children are similar, the rate for Pakistanis is almost twice as high.

### *Associated malformations*

Tables III and IV show associated congenital malformations noted in the children with cancer in Muslim and non-Muslim groups respectively. Malformations were recorded in 21 out of 101 Muslim children, and 6 out of 86 non-Muslim children. Details of these cases are given in the appendix. The rate of malformations (95% confidence intervals shown in brackets) in Muslims was 20.8%, (12.9–28.7), compared with 7% (1.6–12.4) in non-Muslims. The difference in the percentages was 13.8 (4.2–23.4). The malformation rate was significantly ($P<0.01$) higher in Muslims.

The proportion of cancer cases with sporadic genetic disorders was similar in both groups; 8% (8/101) in Muslims and 6% (5/86) in non-Muslims. Among the Muslims, eight cases had autosomal recessive and five autosomal dominant disorders, while one non-Muslim child had an autosomal dominant disorder. Recessive and dominant disorders were therefore found in 13% of Muslims compared with 1% of non-Muslims ($P=0.002$). Two of the dominant disorders in the Muslims may have arisen *de novo* as there was no history of a similarly affected family member.

Tables III and IV also indicate the occurrence of childhood cancer in other close family members. One-third (7/21) of the Muslims with malformations had a close relative with childhood cancer who had a similar congenital abnormality. None of the non-Muslims with malformations had an affected relative with childhood cancer ($P<0.001$).

**Table I** Country of origin and religion of 187 Asian children with cancer

| *Country* | *No. of patients* | *(%)* | *Religion* | *No. of patients* | *(%)* |
|---|---|---|---|---|---|
| India | 84 | 45 | Muslim | 5 | 6 |
| | | | Hindu | 21 | 25 |
| | | | Sikh | 57 | 68 |
| | | | Christian | 1 | 1 |
| Pakistan | 83 | 44 | Muslim | 83 | 100 |
| Bangladesh | 10 | 5 | Muslim | 10 | 100 |
| East Africa | 5 | 3 | Hindu | 4 | 80 |
| | | | Sikh | 1 | 20 |
| Not known | 5 | 3 | Muslim | 3 | 60 |
| | | | Hindu | 1 | 20 |
| | | | Sikh | 1 | 20 |

**Table II** Age-standardised incidence rates (ASRs) and standardised rate ratios (SRRs) for childhood cancer in Asian and white children resident in the West Midlands Region 1978–92

| | Cancer Incidence | | | | | | Rate Ratios | | | | | |
|---|---|---|---|---|---|---|---|---|---|---|---|---|
| | Indians and East Africans | | Pakistanis and Bangladeshis | | White caucasians | | Indian vs. white | | Pakistani vs white | | Pakistani vs Indian | |
| | *Patients* | *ASR* | *Patients* | *ASR* | *Patients* | *ASR* | *SRR* | *(95% CI)* | *SRR* | *(95% CI)* | *SRR* | *(95% CI)* |
| Leukaemia | 32 | 40.9 | 26 | 44.0 | 538 | 43.2 | 0.95 | (0.67–1.34) | 1.02 | (0.68–1.51) | 1.08 | (0.64–1.81) |
| Lymphoma | 17 | 20.5 | 15 | 25.6 | 132 | 9.0 | 2.28 | (1.12–4.64)* | 2.85 | (1.25–6.50)* | 1.25 | (0.62–2.54) |
| CNS tumours | 14 | 16.6 | 18 | 30.8 | 407 | 30.7 | 0.54 | (0.36–0.81)* | 1.00 | (0.62–1.61) | 1.86 | (0.90–3.86) |
| Solid tumours | 28 | 37.5 | 37 | 62.9 | 531 | 42.5 | 0.88 | (0.61–1.26) | 1.48 | (0.996–2.2) | 1.68 | (1.02–2.78)* |
| Sarcomas | 6 | 6.7 | 4 | 6.7 | 179 | 12.9 | 0.52 | (0.28–0.96)* | 0.52 | (0.25–1.07) | 1.00 | (0.28–3.55) |
| Other solid tumours | 22 | 30.7 | 33 | 56.2 | 352 | 29.6 | 1.04 | (0.67–1.61) | 1.90 | (1.19–3.03)* | 1.83 | (1.06–3.17)* |
| All malignancies | 91 | 115.4 | 96 | 163.3 | 1608 | 125.5 | 0.92 | (0.75–1.13) | 1.30 | (1.03–1.64)* | 1.42 | (1.06–1.90)* |

*Significant, $P<0.05$.

Furthermore four Muslims and three non-Muslims without recorded malformations had a family history of childhood cancer. Among the Muslims, a retinoblastoma case had a sibling with a brain tumour and a second cousin with neuroblastoma. A second retinoblastoma case had a sibling with Hodgkin's disease. Two patients with neuroblastoma and acute myelogenous leukaemia (AML) both had first cousins with respectively a CNS tumour and neuroblastoma. Among the non-Muslims, a child with Wilms' tumour had a first cousin with leukaemia, and in another family two siblings had bilateral retinoblastoma (as did their father). In total, 11% of Muslim and 3.5% of non-Muslim cancer patients had a family history of childhood cancer ($P = 0.06$). In 9% of Muslims, first-degree relatives were affected; in 7% the cancer occurred in association with malformations.

Many of the observed malformations were recognised cancer-malformation or predisposition syndromes. In our series, 10% (10/101) of the Muslim children had cancer malformation syndromes compared with 2% of non-Muslims (2/86, $P<0.05$). In the Muslims, three of the eight autosomal recessive conditions and all five dominant disorders were cancer-malformation syndromes. In total, cancer-malformation/predisposition syndromes accounted for nearly half (10/21) of the abnormalities found in Muslims.

### Discussion

Although it forms the largest ethnic minority group in the UK, the Asian community is not a culturally homogeneous group, a fact that has not been addressed in previous studies of childhood cancer in Asians (Stiller *et al.*, 1991; Powell *et al.*, 1994). Dividing the Asians into Indians (non-Muslims) and Pakistanis/Bangladeshis (Muslims) may give a much clearer picture of the epidemiology, despite the reduction in numbers in the study groups.

The incidence rates presented here, and the tests of statistical significance, must be interpreted cautiously since two of the three sets of population figures employed are survey-based estimates, which are thought to underestimate the ethnic minorities (Haskey, 1991). Furthermore, the 1991 census figures may also underestimate the ethnic minority population (Carr-Hill, 1993). However, comparisons with other sources of childhood population data suggest that the undercount from the census does not exceed 10% (Powell, 1994) and is probably much lower. Within the Asian groups, there is some evidence that under-enumeration may be marginally greater for Pakistanis and Bangladeshis than for Indians, but the differences are very slight (OPCS Census ethnic group volumes, forthcoming). The 1981 Labour Force Survey figures compare well (less than 2% difference) with figures from the 1981 census grouped according to country of birth of the head of household (our own unpublished observations). In the absence of more accurate population data these rates are presented as the best available estimates of the true incidence.

Both Asian groups show a higher incidence of lymphoma than the white group. One interpretation of this finding is that Indians and Pakistanis in the West Midlands share common genes, which may increase susceptibility to this disease. Environmental factors, such as overcrowding (which may affect the pattern of childhood infection with Epstein–Barr virus) could also play a part. In Britain, households where children reside with three or more adults account for 21% of Indian, 27% of Pakistani and 5% of white households (Owen, 1993). However, patterns of childhood infection are also thought to be important in the aetiology of leukaemia (Kinlen, 1990). The similarity of the leukaemia rates found in all three groups perhaps casts doubt on whether overcrowding could be an aetiological factor.

The higher incidence of some types of tumour in the Pakistani population compared with the Indian, however, suggests that Pakistani cultural and lifestyle factors (including consanguinity) may play an important role in the aetiology of cancers in these children. The socioeconomic

Table III Congenital abnormalities in Muslim Asian children

| Patient no. | Tumour | Genetic condition | History of abnormality in first degree relative | History of abnormality and cancer in first degree relative | Cancer-malformation/predisposition syndrome | Parents consanguineous | Genetic type |
|---|---|---|---|---|---|---|---|
| 1[b] | Medulloblastoma | Pigmented skin lesions and tumour predisposition | Yes | Yes | Yes[a] | Yes | AR |
| 2[b] | ALL | Pigmented skin lesions and tumour predisposition | Yes | Yes | Yes[a] | Yes | AR |
| 3[c] | Wilms' tumour | Congenital aortic valve disease | Yes | Yes | No? | Yes | AR |
| 4[c] | ALL | Ptosis, hydrocele, inguinal hernia | Yes | Yes | No? | Yes | AR |
| 5 | Hodgkin's disease | Immunodeficiency | Yes | No | No | NK | AR |
| 6 | Wilms' tumour | Severe mental retardation with hypotonia β-thalassaemia trait | Yes | No | No | Yes | AR |
| 7 | Bilateral Wilms' tumour | Mental retardation Microcephaly | No | No | No | Yes | AR |
| 8 | Hepatocellular carcinoma | Tyrosinaemia type I Hypertrophic obstructive cardiomyopathy (HOCM) | No | No | Yes | Yes | AR |
| 9[d] | Glioblastoma | Tuberous sclerosis | Yes | Yes | Yes | Yes | AD |
| 10[d] | T-NHL | Tuberous sclerosis | Yes | Yes | Yes | Yes | AD |
| 11[e] | Medullary carcinoma | MEN 2B | No | No | Yes | NK | AD (new mutation) |
| 12[e] | Medullary carcinoma | MEN 2B | Yes | Yes | Yes | No | AD |
| 13 | Optic glioma | Neurofibromatosis | No | No | Yes | No | AD (new mutation) |
| 14 | Hodgkin's disease | Ichthyosis | – | No | No | No | Sporadic |
| 15 | Rhabdomyosarcoma | Craniostenosis | Yes | No | No | Yes | Sporadic (AR?) |
| 16 | Wilms' tumour | Sensorineural deafness | – | No | No | No | Sporadic |
| 17 | NHL | Hypospadias | – | No | No | NK | Sporadic |
| 18 | Neuroblastoma | Cryptorchidism | – | No | No | NK | Sporadic |
| 19 | Hodgkin's disease | Bilateral squint | – | No | No | No | Sporadic |
| 20 | ALL | Down's syndrome | – | No | Yes | NK | Sporadic |
| 21 | Congenital leukaemia | Down's syndrome | – | No | Yes | Yes | Sporadic |

[a] Syndrome not previously described. [b,c,d] Patients are siblings. [e] Patients are mother and daughter. AR, autosomal recessive; AD, autosomal dominant; NK, not known.

Table IV Congenital abnormalities in non-Muslim Asian children

| Patient no. | Tumour | Genetic condition | History of abnormality in first degree relative | History of abnormality and cancer in first degree relative | Cancer-malformation/predisposition syndrome | Parents consanguineous | Genetic type |
|---|---|---|---|---|---|---|---|
| 21 | Hodgkin's disease | Marfan's syndrome | Yes | No | No | No | AD |
| 22 | Wilms' tumour | WAGR | – | No | Yes | No | Sporadic |
| 23 | ALL | Down's syndrome | – | No | Yes | No | Sporadic |
| 24 | ALL | Cryptorchidism | – | No | No | NK | Sporadic |
| 25 | Hodgkin's disease | Solitary kidney | – | No | No | NK | Sporadic |
| 26 | Ewing's sarcoma | Spina bifida | – | No | No | NK | Sporadic |

AD, autosomal dominant; NK, not known.

circumstances of these two groups are very different (Bundey *et al.*, 1990) and this may have contributed to the different cancer patterns. However, for the tumours in which environmental factors are thought to be particularly important (lymphomas (Stiller and Parkin, 1990) and leukaemias (Draper *et al.*, 1991)), incidence rates were similar in the Indians and Pakistanis. While an investigation of environmental factors was beyond the scope of the present study, these arguments suggest that genetic factors may in fact be more important in explaining the Muslim cancer excess.

The finding that Muslim cancer patients have more malformations than non-Muslims is not unexpected, since congenital abnormalities are known to be more common in Birmingham Pakistani Muslims. Serious congenital abnormalities (defined as those causing death or chronic disability or which require surgical correction or splinting) have been shown to occur in 3.6% of Birmingham-born white children, 2.3% of Indians and 6.6% of Pakistani/Bangladeshis. Furthermore, 3.7% of Pakistani/Bangladeshi babies suffer from autosomal recessive conditions (Bundey and Alam 1993). The difference between the malformation rates in Indians and Pakistanis (4.3%) does in fact lie just within the lower 95% confidence limit for the difference (13.8%, 95% CI 4.2–23.4) found in our study. While it could be argued that a cohort of Birmingham-born Asians may not be an appropriate control group for our study, which covers the whole of the West Midlands Region, there are other factors that suggest strongly that the Muslim cancer excess has a predominantly genetic component and is linked to malformations.

Firstly, there is the finding of a link between familial cancers and genetic abnormalities in the Muslims. In a third of the Muslim cancer patients with abnormalities there was a close relative (sibling or parent) with a childhood cancer and a similar type of abnormality. In 11% of Muslim cancer patients there was a family history of childhood cancer, compared with 3.5% of non-Muslims. Although this difference was only marginally significant, note that two of the non-Muslim cases were of familial retinoblastoma, for which the mechanisms of heredity are well known (Vogel, 1979). In all the other cases, as yet unidentified genes may predispose to the development of cancer in these families. When malformations are present, this suggests that the same genes may be causing both the malformation and the cancer. Secondly, many of the abnormalities seen are recognised cancer-malformation syndromes. These syndromes are significantly more common in Muslim cancer patients (10%) than in non-Muslims (2%).

An earlier UK-based case–control study of cancer and congenital abnormalities (Mann *et al.*, 1993), where 93% of the respondents were white, found that 11% of cases and 5% of controls had congenital abnormalities but the majority (92%) of these conditions were sporadic. Familial childhood cancers were rare (0.4% of cases, 3% of malformations), as were cancer malformation syndromes (1.1% of cases, 10% of malformations). Our figures for non-Muslim Asian children do not differ significantly from these reported values for white children but the pattern of malformations in Muslims is clearly different. This reinforces our assertion that in Muslims, the excess of cancer and of genetic abnormalities is linked, and that the abnormal genes that give rise to both conditions have been inherited, the responsible mutations probably having occurred in an earlier generation.

Application of the age-specific cancer incidence rates for Indians to the Pakistani population reveals that there were 28 more cases among the Muslims than expected. Known cancer-malformation/predisposition syndromes were present in ten Muslims, and in a further six cases a sibling also had cancer. This suggests that around half of the Pakistani cancer excess may be attributable to genetic factors. However, it is predominantly in the solid and CNS tumours that Pakistanis show differences from the Indians, and CNS and solid tumours account for only 57% (12/21) of the malformation-linked tumours (69% among the dominant and recessive malformations). It may be that while genetic factors contribute to the overall tumour excess in Muslims, other, possibly environmental, factors are responsible for the particular distribution of cancer types.

Parental consanguinity has been implicated as causing 60% of the mortality and severe morbidity in Pakistani children in Birmingham, autosomal recessive disorders affecting 3.7% of children (Bundey and Alam, 1993). The fact that a significantly higher ($P<0.05$) proportion of cancer patients (8%) had autosomal recessive disorders provides evidence that consanguinity also increases the risk of cancer. The fact that five of the eight recessive conditions were either familial cancers or known cancer-malformation syndromes adds weight to this argument.

What consanguinity cannot easily explain is the presence of so many autosomal dominant conditions, rare in the general population, whose presence in our study was closely linked to the development of the tumours. The occurrence of some autosomal dominant conditions has been related to higher paternal age at conception (Murdoch *et al.*, 1972; Jones *et al.*, 1975), in the current generation, or in previous generations. High paternal age is frequent in Pakistani families because of the large number of pregnancies (Bundey *et al.*, 1990). However, among the conditions present in our patients, neither neurofibromatosis nor tuberous sclerosis has yet been found to be related to older paternal age at conception. Moreover, in the two cases where the condition appeared to be a new mutation, the fathers were aged under 35 years at conception.

It is recognised that the present study has several shortcomings. Examination of hospital records alone is unlikely to give a complete picture of family health. The lack of a direct control group also makes interpretation of the results difficult. Nevertheless, the unusual pattern of cancer-linked malformations in the Muslim cases indicates that this group requires further study. In practice, questionnaire-based case–control studies often fail to include sufficient ethnic minority patients because of language difficulties. A case–control study of UK Asians is required in order to determine the extent to which consanguinity, paternal age and environmental factors are responsible for the cancer excess in Muslims.

### Conclusions

The incidence of childhood cancer in West Midlands Pakistani children is higher than that seen in Indian or white children. These Pakistani children also show an excess of congenital abnormalities caused by autosomal recessive and dominant genes. Consanguineous marriages and a high paternal age at conception are likely to have contributed to the cancer excess in this group.

We recommend that future research into the patterns and incidence of childhood cancer, and other illnesses in Asian children, should accurately record ethnic origin, religion, parental age and consanguinity since, as our study shows, the cultural and genetic differences between Muslim and non-Muslim may have a significant impact on disease susceptibility.

**Acknowledgements**
The West Midlands Regional Children's Tumour Research Group has received financial support from the West Midlands Regional Health Authority and the Special Trustees of the Former United Birmingham Hospitals Trust Funds.

### References

BOYLE P AND PARKIN DM. (1991). Statistical Methods for Registries. In *Cancer Registration: Principles and Methods*, Jensen OM, Parkin DM, MacLennan R, Muir CS, Skeet RG (eds.) pp. 126–158. IARC Scientific publications no. 95, IARC: Lyon.
BUNDEY S AND ALAM H. (1993). A five-year prospective study of the health of children in different ethnic groups, with particular reference to the effect of inbreeding. *Eur. J. Hum. Genet.*, **1**, 206–219.
BUNDEY S, ALAM H, KAUR A, MIR S AND LANCASHIRE RJ. (1990). Race, consanguinity and social features in Birmingham babies: a basis for prospective study. *J. Epidemiol. Community Health*, **44**, 130–135.
BUNDEY S, ALAM H, KAUR A, MIR S AND LANCASHIRE R. (1991). Why do UK-born Pakistani babies have high perinatal and neonatal mortality rates? *Pediatr. Perinat. Epidemiol.*, **5**, 101–114.
CARR-HILL RA. (1993). Underenumeration in the 1991 census: census inaccurate for many ethnic groups. *Br. Med. J.*, **307**, 1563–1564.
DRAPER GJ, VINCENT TJ, O'CONNOR CM AND STILLER CA. (1991). Socio-economic factors and variations in incidence rates between County Districts. In *The Geographical Epidemiology of Childhood Leukaemia and Non-Hodgkin Lymphomas in Great Britain. 1966–83*. Draper G (ed.) pp. 37–45. HMSO: London.
GOODMAN MT, YOSHIZAWA CN AND KOLONEL LN. (1989*a*). Incidence trends and ethnic patterns for childhood leukaemia in Hawaii: 1960–1984. *Br. J. Cancer*, **60**, 93–97.
GOODMAN MT, YOSHIZAWA CN AND KOLONEL LN. (1989*b*). Ethnic patterns of childhood cancer in Hawaii between 1960 and 1984. *Cancer*, **64**, 1758–1763.
HASKEY J. (1991). The ethnic minority populations resident in private households – estimates by county and metropolitan districts of England and Wales. *Popul. Trends*, **63**, 22–35.
HENLEY A. (1979). *Asian Patients in Hospital and at Home*. Pitman Medical: London.

JONES KL, SMITH DW, HARVEY MAS, HALL BD AND QUAN L. (1975). Older paternal age and fresh gene mutation: data on additional disorders. *J. Pediatr.*, **86**, 84–88.

KINLEN LJ, CLARKE K AND HUDSON C. (1990). Evidence from population mixing in British New Towns 1946–85 of an infective basis for childhood leukaemia. *Lancet*, **336**, 577–582.

MCKUSICK VA. (1978). *Mendelian Inheritance in Man*, 5th edn. The John Hopkins University Press: Baltimore.

MANN JR, DODD HE, DRAPER GJ, WATERHOUSE JAH, BIRCH JM, CARTWRIGHT RA, HARTLEY AL, MCKINNEY PA AND STILLER CA. (1993). Congenital abnormalities in children with cancer and their relatives: results from a case–control study (IRESCC). *Br. J. Cancer*, **68**, 357–363.

MILLER RW, FRAUMENI JF AND MANNING MD. (1964). Association of Wilms' tumour with aniridia, hemihypertrophy and other congenital malformations. *N. Engl. J. Med.*, **270**, 922–927.

MUIR KR, PARKES SE, MANN JR, STEVENS MCG AND CAMERON AH. (1992). Childhood cancer in the West Midlands: incidence and survival 1980–1984, in a multi-ethnic population. *Clin. Oncol.*, **4**, 177–182.

MURDOCH JL, WALKER BA AND MCKUSICK VA. (1972). Parental age effects on the occurrence of new mutations for the Marfan syndrome. *Ann. Hum. Genet.*, **35**, 331–336.

OPCS (OFFICE OF POPULATION CENSUSES AND SURVEYS). (1982). *Labour Force Survey 1981*. HMSO: London.

OPCS (OFFICE OF POPULATION CENSUSES AND SURVEYS). (1992). *Provisional Mid-1991 Population Estimates for England and Wales and Constituent Local and Health Authorities Based on 1991 Census Results*. OPCS Monitor PP1 92/1. HMSO: London.

OWEN D. (1993). *Ethnic Minorities in Great Britain: Housing and Family Characteristics*. 1991 Census statistical paper no. 4. University of Warwick Centre for Research in Ethnic Relations: Warwick, UK.

PARKIN DM, STILLER CA, DRAPER GJ, BIEBER CA, TERRACINI B AND YOUNG JL. (EDS). (1988). *International Incidence of Childhood Cancer*. IARC Scientific Publications No. 87. IARC: Lyon.

POWELL JE, PARKES SE, CAMERON AH AND MANN JR. (1994). Is the risk of cancer increased in UK Asians? *Arch. Dis. Child.*, **71**, 398–403.

RUYMANN FB, MADDUX HR, RAGAB A, SOULE EH, PALMER N, BELTANGADY M, GEHAN EA AND NEWTON WA. (1988). Congenital anomalies associated with rhabdomyosarcoma: an autopsy study of 115 cases. A report from the Intergroup Rhabdomyosarcoma Study Committee. *Med. Pediatr. Oncol.*, **16**, 33–39.

SENIOR PA AND BHOPAL R. (1994). Ethnicity as a variable in epidemiological research. *Br. Med. J.*, **309**, 327–330.

STILLER CA AND PARKIN DM. (1990). International variations in the incidence of childhood lymphomas. *Paediatr. Perinat. Epidemiol.*, **4**, 303–324.

STILLER CA, MCKINNEY PA, BUNCH KJ, BAILEY CC AND LEWIS IJ. (1991). Childhood cancer and ethnic group in Britain: a United Kingdom Children's Cancer Study Group (UKCCSG) Study. *Br. J. Cancer*, **64**, 543–548.

VOGEL F. (1979). Genetics of retinoblastoma. *Hum. Genet.*, **52**, 1–54.

### Appendix

*Malformations and cancer syndromes in Asians. (Cases as listed in Tables III and IV)*

### Muslims

*Patients 1 and 2*

In this Pakistani family, three of the four siblings had pigmented skin lesions and the eldest two siblings had cancer (ALL at the age of 6 years and medulloblastoma at age 10). Their parents were double first cousins and their father had cancer of the ascending colon diagnosed when he was 44 years old. There are several possible genetic explanations for the cluster of cases within this family. This may be a recessive condition, with a late-onset tumour in the father, who is heterozygous for the underlying mutation. However, the family structure does not exclude a dominant condition with variable expression. The family does not meet the diagnostic criteria for neurofibromatosis type I, but shows some similarity to Turcot's syndrome with colonic and cerebral malignancies, infrequent café-au-lait spots and probable recessive inheritance, although to date polyposis coli has not been detected.

*Patients 3 and 4*

It is known that Wilms' tumour is associated with various congenital abnormalities (e.g. WAGR) (Miller *et al*., 1964). Patient 3, aged 11 months, had unilateral Wilms' tumour and congenital aortic valvular disease, while his brother, patient 4, who developed ALL at the age of 2, had hydrocoele, inguinal hernia and unilateral ptosis. A third sibling died of cyanotic congenital heart disease in the neonatal period. The Pakistani parents were first cousins.

*Patient 5*

This Pakistani boy was immunodeficient, and had chronic suppurative lung disease; his brother was similarly affected, though less severely. A third sibling had died in the neonatal period, possibly as a result of an undiagnosed immunodeficiency. The index patient may have developed Hodgkin's disease (at age 10) because of his underlying immunodeficiency. He later relapsed and underwent a bone marrow transplant, but at the age of 15 he developed AML and died soon afterwards. It is not known whether his parents were related.

*Patient 6*

This 2-year-old Pakistani boy had a unilateral Wilms' tumour and severe mental retardation with hypotonia. His sister had a similar neurological disorder and deafness. Both siblings had a $\beta$-thalassaemia trait, also an autosomal recessive condition. There was one normal child in the family; the parents were first cousins.

*Patient 7*

The index patient, a Pakistani girl aged 5 months, had bilateral Wilms' tumours, associated with severe psychomotor retardation, microcephaly, epilepsy and rickets. There was one other normal child in the family and the parents were first cousins. Despite the lack of an affected sibling, the clinical phenotype was highly suggestive of a recessive condition.

*Patient 8*

This 4-year-old Indian patient received a liver transplant for cirrhosis related to tyrosinaemia type I, and hepatocellular carcinoma was discovered in the resected liver. He also had hypertrophic obstructive cardiomyopathy (HOCM), the aetiology of which was unclear. His two siblings were normal. The parents were first cousins, as were both sets of grandparents. Tyrosinaemia is a recognised recessive condition.

*Patients 9 and 10*

Patient 9, who had tuberous sclerosis with severe mental retardation and absence of the corpus callosum, developed a glioblastoma of the posterior fossa at the age of 2 and died within 4 months of diagnosis. His brother, patient 10, also had tuberous sclerosis and developed T-cell non-Hodgkin's lymphoma (NHL) at the age of 3 years, dying within 7 months. His sister and mother also showed signs of tuberous sclerosis. The Pakistani parents were first cousins.

*Patients 11 and 12*

In patient 11 multiple endocrine neoplasia (MEN) II was diagnosed at the age of 13 and at 14 she developed medullary thyroid carcinoma and underwent a total thyroidectomy. There were two healthy siblings, and no evidence of MEN II was found in relatives, although her father died of myocardial infarction at the age of 30. It is not known if her Pakistani parents were related.

Patient 12 was the daughter of patient 11. She underwent total thyroidectomy at the age of 2 for medullary thyroid carcinoma, at which time she had the prominent facies of evolving MEN IIB. She was an only child and her parents were unrelated.

*Patient 13*

This Pakistani boy was diagnosed as having optic nerve glioma at 22 months of age. Multiple café-au-lait spots were noted at the time of diagnosis. There were two healthy older siblings, and there was no evidence of neurofibromatosis or malignant disease in other members of the extended family. The parents were unrelated.

*Patient 14*

This Pakistani boy, who developed Hodgkin's disease at the age of 12, had congenital icthyosis. He was the youngest of six siblings (all healthy) and the father was aged 52 at the child's conception. The parents were unrelated. Some forms of icthyosis are autosomal dominant but there was insufficient evidence to classify this condition as dominant.

*Patient 15*

This Pakistani boy with craniostenosis developed rhabdomyosarcoma of the nasopharynx at the age of 5. Rhabdomyosarcomas have been reported to be associated with malformations of various sytems (Ruymann *et al.*, 1988). He had four siblings who died in early childhood with an undiagnosed neuro-degenerative disorder, but he has not yet manifested any signs of neurodegeneration. Four other siblings were normal. The parents were first cousins.

*Patient 16*

This Pakistani girl presented at 9 years of age with Wilms' tumour. She had had profound bilateral sensorineural deafness since infancy. She had four healthy siblings and her parents were unrelated.

*Patient 17*

This Pakistani boy, one of twins, had hypospadias requiring surgery. He developed T-cell NHL at the age of 5.

*Patient 18*

Stage I neuroblastoma was found in this 6-month-old Pakistani boy during investigations for bilateral undescended testes. He had persistent Müllerian duct syndrome, with normal chromosomes. The tumour regressed without treatment.

*Patient 19*

This Pakistani boy, who developed Hodgkin's disease at the age of 9, had severe bilateral convergent strabismus, requiring surgical correction. His seven siblings were healthy. The parents were unrelated.

*Patient 20*

This Bangladeshi girl had Down's syndrome and Fallot's tetralogy, right microphthalmia and congenital dislocation of the right hip. At 2 years she developed ALL (common). Her two siblings were healthy.

*Patient 21*

This Pakistani girl had Down's syndrome and Fallot's tetralogy and developed congenital megakaryoblastic leukaemia at 19 days. She was the only child of related (first cousin) parents.

**Non-Muslims**

*Patient 22*

This Indian Sikh boy, who developed Hodgkin's disease at 6 years, had Marfan's syndrome with aortic root dilatation and mitral valve prolapse, as well as $\beta$-thalassaemia trait. His father, sister and paternal uncle also had Marfan's syndrome.

*Patient 23*

This Indian Hindu boy, who developed bilateral Wilms' tumour at 18 months, had the WAGR syndrome (Wilms' tumour, aniridia, genitourinary malformations, mental retardation) associated with a deletion on the short arm of chromosome 11. He was an only child.

*Patient 24*

This Indian Sikh boy with Down's syndrome developed pre-B ALL at the age of 5. He had two healthy siblings.

*Patient 25*

This Indian Sikh boy had bilateral undescended testicles, requiring a left orchidopexy at 11 months. His chromosomes were normal. At 2 years he developed common ALL. He had one healthy brother.

*Patient 26*

At the age of 6, this Indian Sikh girl developed Hodgkin's disease of the neck. At the age of 11, investigation of a left sided abdominal mass revealed a missing right kidney, with compensatory hypertrophy of the left kidney.

*Patient 27*

This Indian Hindu boy developed a pelvic primitive neuroectodermal tumour at the age of 5. During investigations spina bifida occulta of the lumbar region was found. He was an only child.